# The Bréant Prize

**Published:** 1858-09

**Authors:** 


THE BRÉANT PRIZE.—It will be remembered that the late Mr. Bréant left a reward of £4000 to the discoverer of an undoubted remedy for, or prophylactic of, cholera. The Academy of Sciences, Paris, which has the supervision of the subject, has lately reviewed the report of the committee appointed to examine the claims of candidates. None of the one hundred and fifty-three essays which were submitted were thought worthy of the prize; but two attracted some attention. The head surgeon of the Smolensk Hospital, Russia, thinking cholera analogous to smallpox and typhoid fever, vaccinates all the patients at the onset of the epidemic. This mode, he claims, saves six out of seven patients. The other writer, who signs himself Mr. R., says that his cures are eighty per cent., and that he uses calomel in two or three-grain doses every two or five minutes, there being no danger of salivation.

